# Low-Intensity Agricultural Landscapes in Transylvania Support High Butterfly Diversity: Implications for Conservation

**DOI:** 10.1371/journal.pone.0103256

**Published:** 2014-07-24

**Authors:** Jacqueline Loos, Ine Dorresteijn, Jan Hanspach, Pascal Fust, László Rakosy, Joern Fischer

**Affiliations:** 1 Institute of Ecology, Leuphana University, Lueneburg, Germany; 2 Organic Agricultural Science Group, University Kassel, Witzenhausen, Germany; 3 Department Taxonomy and Ecology, Babes-Bolay University, Cluj-Napoca, Romania; USDA-Agricultural Research Service, United States of America

## Abstract

European farmland biodiversity is declining due to land use changes towards agricultural intensification or abandonment. Some Eastern European farming systems have sustained traditional forms of use, resulting in high levels of biodiversity. However, global markets and international policies now imply rapid and major changes to these systems. To effectively protect farmland biodiversity, understanding landscape features which underpin species diversity is crucial. Focusing on butterflies, we addressed this question for a cultural-historic landscape in Southern Transylvania, Romania. Following a natural experiment, we randomly selected 120 survey sites in farmland, 60 each in grassland and arable land. We surveyed butterfly species richness and abundance by walking transects with four repeats in summer 2012. We analysed species composition using Detrended Correspondence Analysis. We modelled species richness, richness of functional groups, and abundance of selected species in response to topography, woody vegetation cover and heterogeneity at three spatial scales, using generalised linear mixed effects models. Species composition widely overlapped in grassland and arable land. Composition changed along gradients of heterogeneity at local and context scales, and of woody vegetation cover at context and landscape scales. The effect of local heterogeneity on species richness was positive in arable land, but negative in grassland. Plant species richness, and structural and topographic conditions at multiple scales explained species richness, richness of functional groups and species abundances. Our study revealed high conservation value of both grassland and arable land in low-intensity Eastern European farmland. Besides grassland, also heterogeneous arable land provides important habitat for butterflies. While butterfly diversity in arable land benefits from heterogeneity by small-scale structures, grasslands should be protected from fragmentation to provide sufficiently large areas for butterflies. These findings have important implications for EU agricultural and conservation policy. Most importantly, conservation management needs to consider entire landscapes, and implement appropriate measures at multiple spatial scales.

## Introduction

Almost half of Europe’s terrestrial surface consists of farmland, and many species, including rare and endangered ones, depend on farmland as habitat [1], [2]. The loss of cultural-historic landscapes through intensification or abandonment of farming practices is causing declines of farmland biodiversity [1], [3], [4], [5], [6]. To effectively design conservation strategies, knowledge is needed about which variables influence species richness and distribution at different spatial scales [7], [8], [9].

In Western Europe, species loss in farmland has been associated with an increase of agricultural productivity [9], [10], [11], most likely caused by the use of agrochemicals [12] and the loss and fragmentation of semi-natural patches, especially grasslands [8], [13]. In Eastern Europe, socio-economic conditions and land use have been rapidly changing since the breakdown of communism and accession of new member states to the European Union (EU) [14], [15], [16]. Current changes involve a dual threat to biodiversity, with a trend towards structural simplification on the one hand and abandonment of low-intensity practices on the other hand [17], [18]. The current situation in Eastern Europe thus differs in important ways from Western European countries [1], [19], [20], and a better understanding is needed of how organisms respond to landscape features within low-intensity farming areas of Eastern Europe.

Heterogeneous landscapes typically harbour greater species richness than homogenous landscapes [3], [21], [22], most likely because of their greater niche diversity, as well as spillover effects and habitat complementation [23]. Agricultural simplification and land abandonment typically lead to a loss of landscape connectivity, which may not only dissect the habitats for species, but also causes flow-on effects on the composition and configuration of the landscape as a whole [24], [25].

A particularly interesting cultural-historic region in Eastern Europe is Transylvania, which supports extraordinarily high levels of farmland biodiversity [26], [27]. Especially in its South, Transylvania is characterised by a small-scale mosaic of different low-intensity land-uses that provide many different, well-connected structures such as field margins and roadside vegetation. The historic management of the area has created heterogeneity at multiple spatial scales: within tens of metres (hereafter termed the local scale), in the immediate surroundings around any given location (the context scale), as well as over thousands of metres (the landscape scale) [28], [29].

Here, we focus on butterflies as a taxonomic group that rapidly responds to environmental changes [30] and is known to be sensitive to land use change worldwide [4]. In Europe, many butterflies use anthropogenic landscape elements [31], but species with different traits are expected to respond differently to land use change [8], [32]. For example, Öckinger & Smith [33] found that the effects of landscape composition differed between species of different mobility classes, and Börschig et al. [34] found that intensively used agricultural landscapes mostly support generalists. Yet, evidence on the responses of butterflies to gradients of spatial heterogeneity is sparse, and more thorough studies at multiple scales are needed [22], [35].

We sought to understand the responses of butterfly diversity to key landscape gradients in Southern Transylvania, using a snapshot natural experiment [36], [37] that spanned the full range of environmental conditions with respect to heterogeneity and woody vegetation cover across multiple scales. Our overarching aim was to understand drivers of species richness and composition. Specifically, we asked (i) how landscape structures affect the composition of butterfly communities; (ii) which landscape structures explain butterfly species richness at various spatial scales; and (iii) which landscape structures affect abundance patterns of selected species. We discuss our findings in the context of possible landscape changes that may take place in Transylvania.

## Materials and Methods

### Ethics Statement

We obtained the necessary permit for surveying butterflies within the EU *Natura 2000* network from Progresul Silvic, the organization officially entrusted with the custody of the protected area by the Romanian government. The survey procedure was approved beforehand by the ethics committee of Leuphana University Lueneburg.

### Data Availability Statement

All data underlying the findings reported in this study are available from the Dryad Digital Repository (http://doi.org/10.5061/dryad.97s1k).

### Study area and experimental design

**Figure 1:**
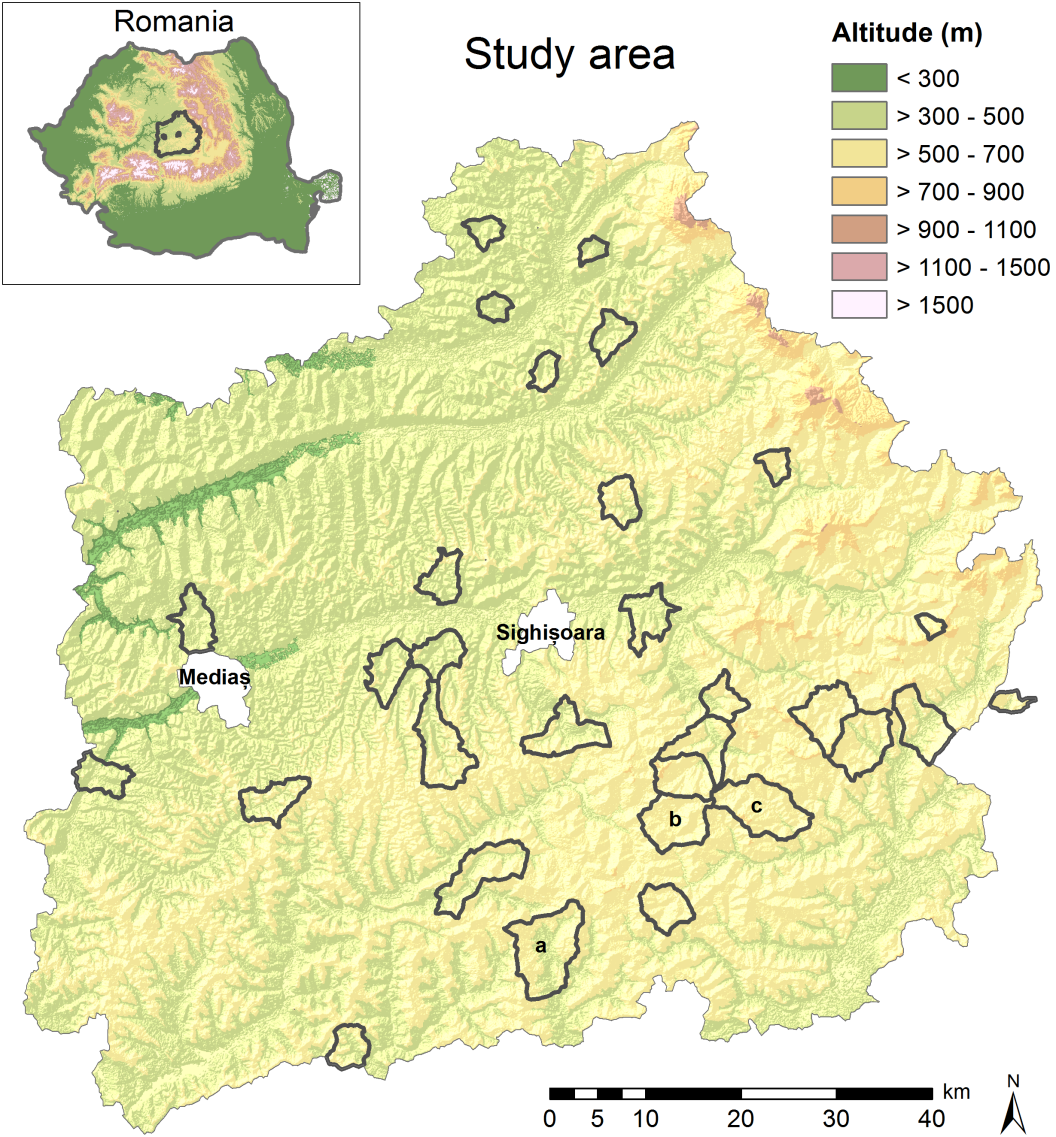
Location of the study area with investigated village catchments in Transylvania, Romania. The small letters indicate the village catchments illustrated for predictions in Figure 4 (a = Cincu, b = Granari, c = Viscri).

The study area covered approximately 7,000 km^2^ in the lowlands of Southern Transylvania, Romania (Figure 1). We followed the notion of a natural experiment [36], with randomised site selection in pre-defined strata at two levels: study villages and survey sites within villages.

To select study villages, we first allocated each raster pixel of the study area to different “village catchments”. These were calculated using a cost-distance algorithm in ARCGIS with the village centre as the reference point and the slope and the distance to the next village as the cost variables. Information about village locations was extracted from CORINE land cover data 2006 (http://www.eea.europa.eu/data-and-maps/data#c12=corine+land+cover+version+13&b_start=0&c17=CLC2006), and slope was calculated from the digital elevation model ASTER (Advanced Spaceborne Thermal Emission and Reflection Radiometer). Topographically based village catchments were used instead of administrative boundaries because administrative boundaries were only available at the commune level (typically 3–5 villages). However, we found that the resulting polygons accurently reflected historical land use responsibilities. Second, we stratified village catchments along a gradient of terrain ruggedness and according to their protection status under the EU Birds and Habitats Directives. Terrain ruggedness was calculated as the standard deviation of the altitude of the catchment, and we used quantiles to classify ruggedness as either low, medium or high. Protection status of the catchments was either unprotected, SCI (Site of Community Importance) or SPA (Special Protection Area). Third, we randomly chose 30 villages, covering all combination of ruggedness and protection status (Table S1).

To select survey sites, we stratified the agricultural area within these 30 villages according to CORINE land cover as grassland or arable land and excluded other land cover classes. Within these strata, we spanned two gradients that we assumed sensitive to change in the future as a result of structural simplification, namely woody vegetation cover and heterogeneity. We estimated woody vegetation cover in a circular one hectare area based on classified 10 m SPOT data (CNES, ISIS programme). To assess heterogeneity, we used the standard deviation of 2.5 m panchromatic SPOT data within a one hectare circle. We assigned each hectare of the agricultural landscape to a combination of three classes of woody vegetation cover by three classes of heterogeneity. We distinguished low (0–5%), medium (>5–15%) and high (>15%) woody vegetation cover and used the lower, middle and upper third of percentiles to classify heterogeneity. Within these combinations, we randomly selected replicates for each cross-combination (except for the combination of high heterogeneity and low woody vegetation cover, which did not exist (Table S2)). In total, we selected 120 circular 1 ha survey sites, with 60 in grassland and 60 in arable land, and an average of four survey sites per village catchment. Notably, sites in arable land in this context were consciously placed not to represent only arable fields specifically, but rather to capture the whole range of conditions within the mosaic of arable land [38], including field margins and fallow land.

### Data collection

#### Butterfly surveys (response data)

We assessed species richness and abundance of butterflies (Rhopalocera) and diurnal burnet moths (Zygaenidae) by walking four transects of 50 m length per survey site [39]. We included burnet moths because they are comparable to butterflies in their ecology [33], [40]. These transect pointed north, east, south and west, and started 6 m from the centre of a given site. In a given transect walk, each butterfly observed within 2.5 m of each side of the transect and 5 m in front of the observer was identified and counted. Species that we could not identify in the field were treated as compound species: *L. sinapis/juvernica*, *C. alfacariensis/hyale* and *Zygaena minos/purpuralis*. *Adscita*, *Jordanita* and *Carcharodus* occurred within the study region, and are represented by two, two and three species, respectively [41]. However, these species are difficult to distinguish and therefore were only identified to the genus level. Surveys were repeated on four occasions between May and August 2012 by four different, trained observers. Surveys were conducted under suitable weather conditions (no rain, <90% cloud cover, >17°C, no strong wind), between 9 am and 5 pm.

#### Environmental data (explanatory variables)

We followed a multi-scale approach and included explanatory variables that could potentially explain butterfly distribution at the local (1 ha), context (50 ha) and landscape scale (i.e. village catchments, ranging from 430 to 4963 ha). An overview of all variables included in the analysis is presented in Table 1.

**Table 1:** Definition of environmental variables used in the study at three different scales and method of obtaining those. Abbreviations are used in Figure 2 and Table 2.

Scale	Variable (abbreviation)	Definition and method
**local (1 ha)**	Number of plantsspecies (NoPlant)	Vascular plant species richness assessed by eight randomly distributed quadrants of one by one meter
	Heterogeneity(het_1 ha)	Heterogeneity measured as the standard deviation of 2.5 m panchromatic SPOT picture (CNES, ISIS programme)
	Woody vegetationcover(woody_1 ha)	Proportion of woody vegetation cover based on classified 10 m SPOT satellite image (CNES, ISIS programme)
	Heat index(heatload)	Potential for ground heating calculated after Parker [42]: Heat index = cos (slope aspect−225) * tan (slope angle)
	Terrain WetnessIndex (TWI)	Measure of potential soil wetness, estimated as the position in the landscape and the slope from ASTER digital elevation model with 30 m resolution.
	Land Cover(LU_type)	Land use classification as arable land, grassland or forest based on CORINE land cover
**context (50 ha)**	Ruggedness(rugg_50 ha)	Terrain ruggedness, calculated as standard deviation of altitude
	Woody vegetationcover (woody_50 ha)	Proportion of woody vegetation cover based on classified 10 m SPOT satellite image
	Configurational heterogeneity(ED_50 ha)	Configuration of different land covers, calculated as the edge density with FRAGSTATS v4.2 based on CORINE land cover
**landscape (village catchment)**	Amount of pasture(past_catch)	Proportion of pasture, based on CORINE land cover
	Woody vegetationcover (woody_catch)	Proportion of forest cover based on CORINE land cover
	Ruggedness(catch_rugg)	Terrain ruggedness, calculated as the standard deviation of the altitude
	Compositionalheterogeneity (SIDI)	Composition of different land covers, calculated as Simpson index of diversity with FRAGSTATS v4.2 based on CORINE land cover
	Configurationalheterogeneity (ED)	Configuration of different land covers, calculated as edge density with FRAGSTATS v4.2 based on CORINE land cover
**Random effects**	Village catchment	Classification of the landscape into social-ecological units according to a cost distance algorithm of proximity to the nearest village as reference point and the slope of the terrain as cost factor
	Level	Observation level random effect

At the local scale, we collected data on vascular plant species richness in eight randomized quadrants (1×1 m). We used cumulative plant species richness per site as an explanatory variable. We also calculated indices for heatload (after [42]) and terrain wetness as a measure of potential soil wetness, and included heterogeneity assessed by the spectral variance of SPOT data (see Table 1 for details). We calculated percent woody vegetation cover at local and context scales, and used CORINE land cover to calculate percent forest at the landscape scale. For the context and landscape scales, we calculated the terrain ruggedness as the standard deviation of altitude. We also quantified compositional or configurational heterogeneity of the different land covers grassland, arable land and forest as provided by CORINE land cover data. At the context scale, our chosen heterogeneity measures (Simpson index of land cover diversity, edge density) were correlated (r = 0.76). Hence, we included only edge density as an explanatory variable (following [7]). At the landscape scale, we used both edge density and the Simpson index of diversity and added the amount of pasture and forest per village catchment, based on CORINE land cover data. Variables on compositional and configurational heterogeneity were calculated using FRAGSTATS v4.2 [43] and all other variables using ARCGIS 10.1 (ESRI Inc., Redland, CA).

### Analysis

We pooled all observed butterfly species and individuals from the four survey rounds for each survey site. First, we tested for differences in species richness and abundance between different levels of official protection by using Analysis of Variance (ANOVA). Second, we conducted a detrended correspondence analysis (DCA) to describe species composition and its relation to environmental variables. We used a permutation test to fit and test the correlation of environmental variables with the ordination.

Third, we used generalized linear mixed effects models (GLMMs) to assess effects of environmental variables on butterfly species richness. Beforehand, we tested the explanatory variables for collinearity (all r<0.7; Table S4; [44]). We log-transformed woody vegetation cover at local and context scales and heterogeneity at the local scale because these variables were highly skewed. All numerical explanatory variables were scaled to mean zero and unit variance. We included the variables listed in Table 1 to model species richness of butterflies. To test for a unimodal relationship in response to woody vegetation cover, we included a quadratic term of local woody vegetation cover. We furthermore expected that the effect of heterogeneity may differ between grassland and arable land and therefore included an interaction term between land cover type and heterogeneity. Grasslands are also interesting to look at separately because they are among the most species rich biotopes for butterflies in Europe [45]. We assessed the variance inflation factor (VIF) of the generalized linear model (GLM) and tested for spatial auto-correlation in the residuals. We included the village catchment as a random effect and corrected for overdispersion by adding an observation level random effect. We simplified the model by stepwise backwards selection retaining all variables with p<0.1. For GLMMs, significance levels are only approximations, hence many statisticians suggest using a significance level of p<0.1 [46].

Likewise, we modelled species richness of functional groups. To this end, we distinguished between species of low mobility (Bink’s mobility classes 1 and 2) and high mobility (Bink’s mobility classes 7, 8 and 9; [47]). Highly mobile species were *Colias crocea*, *Pieris brassicae*, *Vanessa atalanta* and *Vanessa cardui*. Low-mobility species were *Brenthis daphne*, *Brenthis ino*, *Coenonympha glycerion*, *Cupido minimus*, *Euphydryas aurinia*, *Hamaeris lucina*, *Heteropterus morpheus*, *Lopinga achine*, *Melitaea britomartis*, *Melitaea diamina* and *Satyrium acaciae*. As a third group we modelled the richness of grassland specialists, namely *Euphydryas aurinia*, *Polyommatus coridon*, *Cyaniris semiargus*, *Lysandra bellargus*, *Phengaris arion*, *Cupido minimus* and *Erynnis tages* [48].

We also modelled the abundance of individual species considered to be declining in Western and Northern Europe, but that are widespread or even increasing in Eastern Europe [48], [49], [50], [51]. We only used species that were common enough in the study area to obtain reliable models, namely *Maniola jurtina*, *Coenonympha pamphilus*, *Polyommatus Icarus, Lycaena dispar* and *Glaucopsyche alexis.* We performed all statistical analyses in R [52], using the packages MASS, ade4, vegan, gdata and lme4.

## Results

In total, we counted 19,878 individuals of 112 species of butterflies (Table S3). Site-level species richness varied between three and 45, and the number of individuals between seven and 452. Eighty-five percent of all individuals belonged to 12 species: *Colias alfacariensis/hyale, Minois dryas, Aphantopus hyperantus, Pieris rapae, Everes argiades, Coenonympha glycerion, Leptidea sinapis/juvernica, Melanargia galathea, Coenonympha pamphilus, Maniola jurtina, Polyommatus icarus,* and *Plebeius argus*. SCI, SPA and unprotected sites did not differ in species richness (F = 0.54, p = 0.58) but SCI sites appeared to have a slightly lower abundance of individuals than unprotected sites (F = 2.37, p = 0.09). Arable land and grassland did not differ in species richness (F = 1.32, p = 0.25) nor abundance of individuals (F = 1.51, p = 0.22).

**Figure 2:**
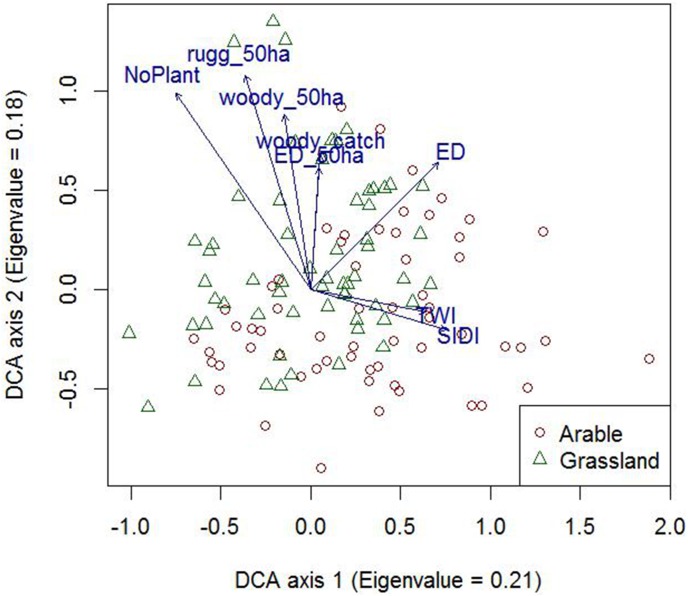
DCA ordination plot of butterfly species, with significant environmental variables superimposed (p<0.05) (Abbreviations: NoPlant = Local plant species richness; TWI = Local terrain wetness index; rugg_50 ha = context terrain ruggedness; woody_50 ha = context woody vegetation cover; ED_50 ha = context edge density; woody_catch = landscape woody vegetation cover; SIDI = landscape compositional heterogeneity; **Table 1** **).**

Multivariate analysis showed substantial overlap in species composition between arable land and grassland (Figure 2), with less than one complete species turnover (length of first axis = 2.9). The first axis (Eigenvalue = 0.21) described a gradient from sites with a low terrain wetness index in homogenous landscapes to sites with a high terrain wetness index within highly heterogeneous landscapes. The second DCA axis (Eigenvalue = 0.18) described a gradient from low to high richness of vascular plants, ruggedness, woody vegetation cover and context-level heterogeneity and landscape-level woody vegetation cover.

**Figure 3:**
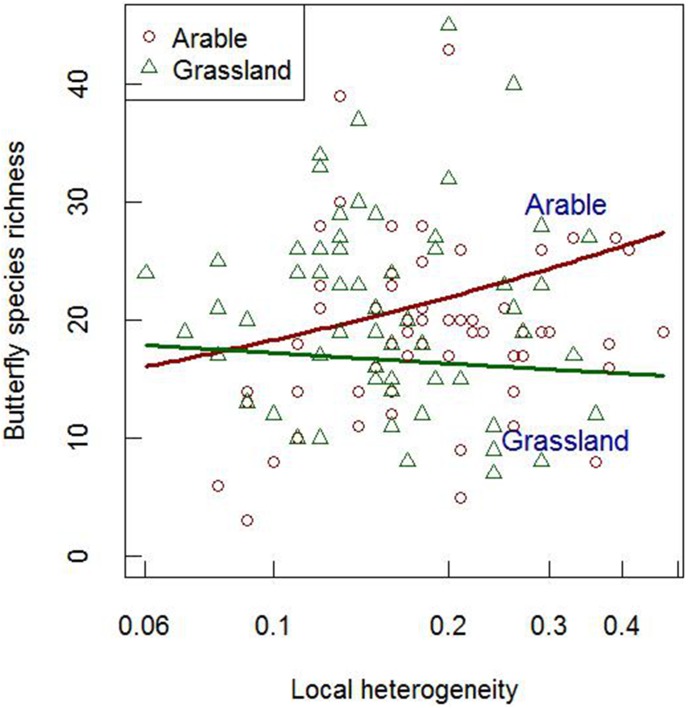
Predicted effect of local heterogeneity on species richness in arable land versus grassland, based on the simplified generalized linear mixed model (**Table 2**).

**Figure 4:**
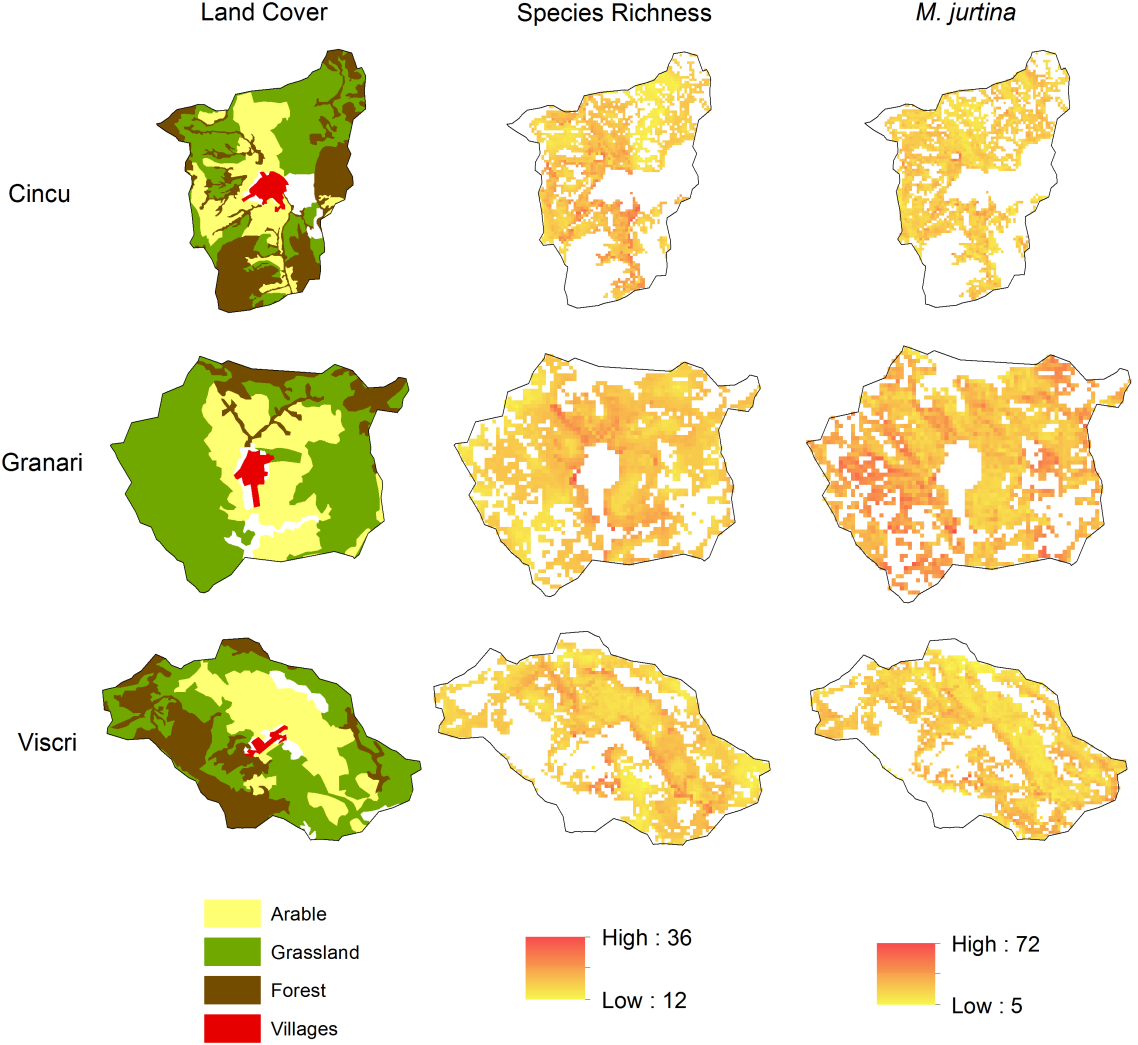
Maps of predicted butterfly distributions in three example villages. Left: Land cover map according to CORINE 2006; middle: predicted species richness for arable and grassland areas within each village catchment; right: predicted abundance of the Meadow Brown (*Maniola jurtina*).

Butterfly species richness was positively related to local plant species richness and local woody vegetation cover, but negatively to local heatload (Table 2). It increased in response to local heterogeneity in arable sites, but not in grasslands (Figure 3). Species richness furthermore increased with configurational heterogeneity and ruggedness at the context scale, but decreased with landscape woody vegetation cover. The models show suitable areas for species of conservation interest exist throughout village catchments, especially in large grassland areas and boundary areas of arable land (Figure 4).

**Table 2:** Parameter estimates of the species distribution models with significance levels indicated by: ^†^P<0.1; *P<0.005; **P<0.01; ***P<0.001.

	SpeciesRichness	High mobilespecies	Low mobilespecies	Specialists	*L. dispar*	*G. alexis*	*P. icarus*	*M. jurtina*	*C. pamphilus*
Intercept	3.026	–0.739	0.681	–0.436	0.581	–1.172	2.914	2.637	1.959
NoPlant	0.261***		0.600***	0.685***	0.581*	0.941**		0.597***	0.313***
LU_type	–0.243**	–1.250***		0.052^†^	–2.059***	–1.554*	–0.040	0.046	0.210
het_1 ha	0.109*						0.278**	0.235*	0.224*
LU_type*het_1 ha	–0.140*						–0.326*	–0.415**	–0.387*
TWI									
woody_1 ha	0.072*		–0.054		0.443^†^				0.102
woody_1 ha∧2			0.232^†^						0.177*
Heatload	–0.057*				–0.622^†^		–0.167*	–0.321***	–0.207**
rugg_50 ha	0.064^†^					0.511*			
woody_50 ha									
ED_50 ha	0.077*		0.261*						
woody_catch	–0.079*							–0.412***	–0.232^†^
past_catch						0.721**	0.256.		
rugg_catch		–0.423*	0.249*	0.051**					
ED			–0.374**						
SIDI		0.448*							

Arable land was used as the baseline land cover in all models. See Table 1 for abbreviations.

Species richness of mobile butterflies was highest in arable land, and responded positively at the landscape scale to both compositional heterogeneity and ruggedness. By contrast, richness of low-mobility species was negatively related to landscape configurational heterogeneity, but responded positively to local-scale plant species richness and context heterogeneity (for additional details, see Table 2). Richness of specialist species was higher in grassland, in landscapes with high terrain ruggedness and at sites with high plant species richness.

For individual species, both *L. dispar* and *G. alexis* were more abundant in arable land, and were positively related to local plant species richness. *L. dispar* also responded positively to local woody vegetation cover, but negatively to local heatload, whereas *G. alexis* showed a positive response to context ruggedness and the amount of grassland in the landscape. The abundances of *P. icarus, M. jurtina* and *C. pamphilus* increased with heterogeneity in arable land, but not in grassland, and decreased with increasing heatload. Abundance of *M. jurtina* and *C. pamphilus* were positively related to local plant species richness, and negatively to landscape woody vegetation cover. *P. icarus* responded positively to the amount of grassland in the landscape. Abundance of *C. pamphilus* was unimodally related to local woody vegetation cover.

## Discussion

We found a high diversity of butterflies in the cultural-historic landscape of Southern Transylvania. This is especially the case considering that we did not seek out sites expected a priori to harbour great diversity, but rather surveyed randomly selected sites within the agricultural matrix. An even greater diversity of butterflies, including rare and endangered species, would be expected to occur in dry grassland patches and traditionally managed hay-meadows, which occur within our study area but which we did not specifically target. Our findings suggest that some types of land use change could pose serious threats to butterfly diversity in Transylvania. Our findings can be summarised within four themes, which we discuss in the following: (i) both grassland and arable land have conservation value; (ii) low-intensity landscapes provide important resources for butterflies; (iii) heterogeneity has a different effect in arable land than in grassland; and (iv) it is important to consider multiple scales for effective butterfly conservation.

### Both grassland and arable land have conservation value

Our findings revealed a high conservation value for butterflies of the small-scale farming system in the lowlands of Transylvania. Interestingly, butterfly species richness and abundance were similar in arable land and grassland. This is a surprising result and suggests a need to broaden the emphasis of conservation activities from grassland protection towards the maintenance of heterogeneous mosaic farmland, including cropland [38]. This is particularly important in the context of criticisms that the recent reform of the European Union’s Common Agricultural Policy, for example, falls far short of what is needed in terms of biodiversity conservation [53]. Throughout Europe, grasslands are considered most important for butterfly conservation (e.g. [8], [45]). Arable land, on the other hand, has received far less attention. In Western Europe, arable land has been found to support lower species richness and more homogenous butterfly communities than grassland [9], [54]. Our results indicate that this situation may be different in Eastern Europe, and that certain types of arable land can in fact support similar levels of butterfly diversity as grasslands. A possible explanation for the similar species richness in arable land and grassland in Transylvania may be spillover effects [23], which may be more likely in small-scale mosaics of land covers. The mosaic character of the landscape also could explain the strong overlap in butterfly communities between arable land and grassland.

### Low-intensity landscapes provide important resources for butterflies

The fine-grained mosaic nature of arable land and the low-intensity nature of grassland in Southern Transylvania emphasize that low-intensity land use practices have major benefits for butterfly conservation. Semi-natural elements occur throughout the landscape, and are a likely reason why species richness is high throughout different land covers [22]. Furthermore, species richness of vascular plants can be high in field margins, which in turn may indicate high quality habitat for butterflies [55]. Consistent with the findings of Kumar, Simonson & Stohlgren [7], we found plant species richness strongly related to butterfly species richness. Currently, Transylvania contains some of the world’s most species rich areas for plants [56], which is partly linked to the low use of fertilizers [57]. Agricultural intensification, by contrast, would likely lead to increased use of fertilizers and hence reduced plant species richness [58], [59], [60]. Furthermore, intensification is typically associated with the use of fewer, high yielding crop varieties. Interestingly, many butterflies in Transylvania use the common crop *Medicago sativa ssp. sativa* (Alfalfa), a leguminous species that provides nectar and that we also observed to serve as a host plant for several butterfly species (e.g. *Glaucopsyche alexis*). Alfalfa is grown in small parcels, is primarily used as winter fodder for livestock, and may easily be lost as a result of intensification. However, high amounts of floral resources are critically important to maintain butterfly diversity. Similarly, woody vegetation offers important resources for butterflies, including shelter and space for thermoregulation [61]. At present, Transylvania contains many scattered trees and hedgerows, and we found that butterfly species richness responded positively to these structures at the local scale. By contrast, a large amount of woody vegetation at the landscape scale may lead to decreased species richness, probably due to a lack of open habitat.

### Heterogeneity has a different effect in arable land than in grassland

We considered heterogeneity and woody vegetation cover at the local scale as two potentially important gradients describing the structure of the landscape. Interestingly, our results showed that the effect of local heterogeneity on species richness depended on land cover. In arable land, species richness increased with heterogeneity, supporting our hypothesis that small-scale farming benefits biodiversity by providing a range of different resources for butterflies. Notably, our land use class of “arable land” reflected the highly heterogeneous nature of traditional farmland, and included cropped areas as well as fallows and uncultivated field margins. These non-cropped areas are likely to be particularly important to maintain butterfly diversity in arable land. By contrast, in grassland, high heterogeneity was associated with reduced butterfly diversity. A possible explanation for this pattern is that heterogeneity of grassland may correspond to a higher degree of fragmentation of butterfly habitat, with likely negative consequences for species diversity [62]. Our study thus confirms that heterogeneity *per se* is not universally beneficial for species richness (see also [63]), although most work to date has focused on its positive effects (e.g. [64]).

### The importance of considering multiple scales

To date, results from studies investigating multiple scales have been disparate and difficult to generalize [65]. We included three spatial scales in our study which we considered relevant for butterfly diversity and distribution. Our study revealed that all investigated scales affected butterfly community composition. Previous studies found local factors affecting butterfly species composition, with local heterogeneity in land cover being a good predictor for species composition in Canada [54], [64]. Butterfly species composition in Transylvania also showed a significant correlation with local factors, but was explained by heterogeneity and woody vegetation cover only at the two larger scales. Butterfly species richness also responded to variables at all different spatial scales, especially at the local scale, but also at the two larger scales (see also [9]). This suggests that local habitat conditions are particularly important, yet these cannot be considered in isolation from the surrounding landscape [33], [66].

Our models also showed that the different functional groups of butterflies were affected by variables from different spatial scales. For example, landscape heterogeneity appeared to benefit mobile species but not low-mobility species. Furthermore, we found that woody vegetation cover was related to species richness. Land abandonment induces natural succession, whereas intensification leads to loss of scattered woody vegetation, and both have negative effects on butterfly richness in the long term [67]. Both processes decrease structural heterogeneity, which is important for viable butterfly populations in agricultural landscapes. In our study, only *Coenonympha pamphilus* showed a unimodal relationship to local woody vegetation cover. For such low-mobile species, presence of woody vegetation is crucial for wind shield and thermoregulation. *C. pamphilus* is abundant in Transylvania, however its population state in other European countries is declining [68]. Habitat heterogeneity from different spatial scales, including the presence of woody vegetation, should be further investigated as possible key elements in landscapes to halt biodiversity loss in farmland.

## Conclusion

Collapse of communism and accession of Romania to the European Union have accelerated land use change in the rural areas of Transylvania, in particular towards land abandonment and agricultural intensification. The two key gradients considered in this study, namely woody vegetation cover and heterogeneity, would fundamentally change as a result of these two land use change processes. Along the gradients of woody vegetation cover and heterogeneity, we were able to show that butterfly abundance and distribution were affected by a range of different variables operating at multiple spatial scales. Not only local conditions, but the composition and configuration of the landscape as a whole need to be considered for effective conservation management of butterflies in low-intensively managed farming landscapes such as in Transylvania.

Our results showed that, unlike in Western Europe, species richness of butterflies was not only high in grassland, but also in arable land. This suggests that more emphasis needs to be placed on low-intensity farming practices and management of the landscape mosaic, and that arable land needs to be actively considered in butterfly conservation strategies. In our study area, butterfly richness would likely benefit from (1) the continuation of small-scale farming; (2) the production of a variety of crops, including legume species; and (3) the maintenance of broad field margins and uncultivated ruderal areas. New payment schemes under the Common Agricultural Policy have recently been criticised as grossly inadequate [53]. Our findings suggest that even measures considered adequate in Western Europe may not be directly transferable to Transylvania – in low-intensity landscapes, it will be particularly important to consider the high nature value that entire agro-ecosystems provide, both inside and outside of protected areas (see also [69]). Ultimately, the continued existence of historic-cultural landscapes such as those in Transylvania hinges on the successful transfer of its appreciation and historic management to future generations of farmers. Substantial efforts are therefore needed in environmental education and in developing alternative ways for local people to make a living, for example through the development of cultural and ecological tourism.

## Supporting Information

Table S1 **Number of the 30 focal villages within different strata.** TRI = Terrain Ruggedness index, Protection status is according to the EU Habitats and Birds Directives.
(DOCX)

Table S2 **Number of survey sites along the two gradients local heterogeneity and local woody vegetation cover.**
(DOCX)

Table S3 **Species list of butterfly species observed in the transects.**
(DOCX)

Table S4 **Correlation matrix of the variables used in the study.**
(DOCX)
