# Supplementary material for: Low-Intensity Agricultural Landscapes in Transylvania Support High Butterfly Diversity: Implications for Conservation
Source: PLoS One. 2014 Jul 24;9(7):e103256. doi: 10.1371/journal.pone.0103256 (PMC4110012; doi:10.1371/journal.pone.0103256)
Supplement: Table S1 — Number of the 30 focal villages within different strata. TRI = Terrain Ruggedness index, Protection status is according to the EU Habitats and Birds Directives. (DOCX) [file pone.0103256.s001.docx]

**Table S1.** Number of the 30 focal villages within different strata. TRI= Terrain Ruggedness index, Protection status is according to the EU Habitats and Birds Directives

|  | **Protection status** | | | |
| --- | --- | --- | --- | --- |
|  |  | No | SCI | SPA |
| **TRI** | Low | 4 | 1 | 4 |
|  | Medium | 3 | 4 | 3 |
|  | High | 4 | 4 | 3 |
