# Supplementary material for: Low-Intensity Agricultural Landscapes in Transylvania Support High Butterfly Diversity: Implications for Conservation
Source: PLoS One. 2014 Jul 24;9(7):e103256. doi: 10.1371/journal.pone.0103256 (PMC4110012; doi:10.1371/journal.pone.0103256)
Supplement: Table S2 — Number of survey sites along the two gradients local heterogeneity and local woody vegetation cover. (DOCX) [file pone.0103256.s002.docx]

**Table S2**. Number of survey sites along the two gradients local heterogeneity and local woody vegetation cover

|  |  | |  | | **Heterogeneity** | | | |  |
| --- | --- | --- | --- | --- | --- | --- | --- | --- | --- |
|  |  | low | | | | medium | | high | |
|  |  | arable land | | grassland | | arable land | grassland | arable land | grassland |
| **Woody vegetation cover** | Low | 8 | | 7 | | 7 | 8 | 0 | 0 |
|  | Medium | 7 | | 8 | | 8 | 7 | 7 | 8 |
|  | High | 8 | | 7 | | 7 | 8 | 8 | 7 |
