# Supplementary material for: Low-Intensity Agricultural Landscapes in Transylvania Support High Butterfly Diversity: Implications for Conservation
Source: PLoS One. 2014 Jul 24;9(7):e103256. doi: 10.1371/journal.pone.0103256 (PMC4110012; doi:10.1371/journal.pone.0103256)
Supplement: Table S3 — Species list of butterfly species observed in the transects. (DOCX) [file pone.0103256.s003.docx]

**Table S3**. Species list of butterfly species observed in the transects

| *Adscita sp* |
| --- |
| *Aglais urticae* |
| *Antocharis cardamines* |
| *Apatura ilia* |
| *Apatura iris* |
| *Aphantopus hyperantus* |
| *Aporia crataegi* |
| *Araschnia levana* |
| *Argynnis adippe* |
| *Argynnis aglaja* |
| *Argynnis laodice* |
| *Argynnis niobe* |
| *Argynnis paphia* |
| *Aricia agestis* |
| *Aricia artaxerxes* |
| *Boloria dia* |
| *Boloria euphrosyne* |
| *Boloria selene* |
| *Brenthis daphne* |
| *Brenthis hecate* |
| *Brenthis ino* |
| *Brinthesia circe* |
| *Cacharodus sp* |
| *Callophrys rubi* |
| *Celastrina argiolus* |
| *Coenonympha arcania* |
| *Coenonympha glycerion* |
| *Coenonympha pamphilus* |
| *Colias alfacariensis/ hyale* |
| *Colias crocea* |
| *Cupido decoloratus* |
| *Cupido minimus* |
| *Cupido osiris* |
| *Cyaniris semiargus* |
| *Erebia medusa* |
| *Erynnis tages* |
| *Eumedonia eumedon* |
| *Euphydryas aurinia* |
| *Everes alcetas* |
| *Everes argiades* |
| *Glaucopsyche alexis* |
| *Gonepteryx rhamni* |
| *Hamearis lucina* |
| *Hesperia comma* |
| *Heteropterus morpheus* |
| *Hipparchia fagi* |
| *Inachis io* |
| *Iphiclides podalirius* |
| *Issoria lathonia* |
| *Jordanita sp* |
| *Lasiommata megera* |
| *Leptidea morsei* |
| *Leptidea sinapis/ juvernica* |
| *Limenitis camilla* |
| *Limenitis populi* |
| *Lopinga achine* |
| *Lycaena alciphron* |
| *Lycaena dispar* |
| *Lycaena phleas* |
| *Lycaena thersamon* |
| *Lycaena tityrus* |
| *Lycaena virgaureae* |
| *Lysandra bellargus* |
| *Phengaris arion* |
| *Maniola jurtina* |
| *Melanargia galathea* |
| *Meleagera daphnis* |
| *Melitaea athalia* |
| *Melitaea aurelia* |
| *Melitaea britomartis* |
| *Melitaea cinxia* |
| *Melitaea diamina* |
| *Melitaea didyma* |
| *Melitaea phoebe* |
| *Melitaea trivia* |
| *Minois dryas* |
| *Neptis sappho* |
| *Nymphalis antiopa* |
| *Ochlodes sylvanus* |
| *Papilio machaon* |
| *Pararge aegeria* |
| *Pieris brassicae* |
| *Pieris napi* |
| *Pieris rapae* |
| *Plebejus argus* |
| *Plebejus argyrognomon* |
| *Plebejus idas* |
| *Polygonia c-album* |
| *Polyommatus amandus* |
| *Polyommatus coridon* |
| *Polyommatus dorylas* |
| *Polyommatus icarus* |
| *Polyommatus thersites* |
| *Pontia edusa* |
| *Pseudophilotes vicrama* |
| *Pyrgus armoricanus* |
| *Pyrgus alveus* |
| *Pyrgus malvae* |
| *Rhagades pruni* |
| *Satyrium acaciae* |
| *Satyrium ilicis* |
| *Thymelicus lineola* |
| *Thymelicus sylvestris* |
| *Vanessa atalanta* |
| *Vanessa cardui* |
| *Zygaena angelicae* |
| *Zygaena carniolica* |
| *Zygaena ephialtes* |
| *Zygaena filipendulae* |
| *Zygaena loti* |
| *Zygaena minos/purpuralis* |
| *Zygaena viciae* |
