# Supplementary material for: Low-Intensity Agricultural Landscapes in Transylvania Support High Butterfly Diversity: Implications for Conservation
Source: PLoS One. 2014 Jul 24;9(7):e103256. doi: 10.1371/journal.pone.0103256 (PMC4110012; doi:10.1371/journal.pone.0103256)
Supplement: Table S4 — Correlation matrix of the variables used in the study. (DOCX) [file pone.0103256.s004.docx]

**Table S4.** Correlation matrix of the variables used in the study

|  | **NoPlant** | **het_ 1ha** | **woody_ 1ha** | **heat-**  **load** | **TWI** | **rugg_ 50ha** | **woody_ 50ha** | **ed_ 50ha** | **past_ catch** | **woody_ catch** | **catch_ rugg** | **SIDI** | **ED** |
| --- | --- | --- | --- | --- | --- | --- | --- | --- | --- | --- | --- | --- | --- |
| **NoPlant** | 1.00 | -0.22 | 0.07 | 0.03 | -0.25 | 0.39 | 0.27 | 0.06 | -0.09 | 0.14 | -0.02 | -0.17 | -0.11 |
| **het_1ha** | -0.22 | 1.00 | 0.22 | 0.15 | 0.09 | 0.02 | 0.09 | 0.12 | -0.18 | 0.15 | 0.03 | 0.02 | 0.11 |
| **woody_1ha** | 0.07 | 0.22 | 1.00 | 0.00 | 0.25 | 0.10 | 0.29 | 0.02 | 0.02 | 0.22 | -0.04 | -0.20 | -0.15 |
| **heatload** | 0.03 | 0.15 | 0.00 | 1.00 | -0.04 | 0.12 | 0.00 | 0.06 | 0.00 | -0.01 | 0.13 | 0.15 | 0.18 |
| **TWI** | -0.25 | 0.09 | 0.25 | -0.04 | 1.00 | -0.13 | -0.02 | 0.02 | -0.05 | 0.17 | 0.10 | 0.04 | -0.04 |
| **rugg_50ha** | 0.39 | 0.02 | 0.10 | 0.12 | -0.13 | 1.00 | 0.49 | 0.27 | -0.29 | 0.38 | 0.24 | -0.07 | 0.15 |
| **woody_50ha** | 0.27 | 0.09 | 0.29 | 0.00 | -0.02 | 0.49 | 1.00 | 0.42 | -0.29 | 0.61 | 0.01 | -0.27 | 0.14 |
| **ed_50ha** | 0.06 | 0.12 | 0.02 | 0.06 | 0.02 | 0.27 | 0.42 | 1.00 | -0.29 | 0.30 | 0.20 | -0.12 | 0.31 |
| **past_catch** | -0.09 | -0.18 | 0.02 | 0.00 | -0.05 | -0.29 | -0.29 | -0.29 | 1.00 | -0.46 | -0.10 | -0.11 | -0.11 |
| **woody_catch** | 0.14 | 0.15 | 0.22 | -0.01 | 0.17 | 0.38 | 0.61 | 0.30 | -0.46 | 1.00 | 0.09 | -0.29 | -0.05 |
| **catch_rugg** | -0.02 | 0.03 | -0.04 | 0.13 | 0.10 | 0.24 | 0.01 | 0.20 | -0.10 | 0.09 | 1.00 | 0.25 | 0.09 |
| **SIDI** | -0.17 | 0.02 | -0.20 | 0.15 | 0.04 | -0.07 | -0.27 | -0.12 | -0.11 | -0.29 | 0.25 | 1.00 | 0.29 |
| **ED** | -0.11 | 0.11 | -0.15 | 0.18 | -0.04 | 0.15 | 0.14 | 0.31 | -0.11 | -0.05 | 0.09 | 0.29 | 1.00 |
